# Draft Genome Sequence of a New Staphylococcal Species Isolated from Human Skin

**DOI:** 10.1128/MRA.01499-19

**Published:** 2020-02-06

**Authors:** Kristian Stødkilde, Anja Poehlein, Holger Brüggemann

**Affiliations:** a Department of Biomedicine, Aarhus University, Aarhus, Denmark; b Department of Genomic and Applied Microbiology, Institute of Microbiology and Genetics, University of Göttingen, Göttingen, Germany; Indiana University, Bloomington

## Abstract

A hemolytic staphylococcal strain, *Staphylococcus* sp. strain 170179, was isolated from healthy human skin. Genome sequencing and comparison of strain 170179 to other staphylococci revealed a relatedness to Staphylococcus haemolyticus with an average nucleotide identity of 87.5%, indicating that *Staphylococcus* sp. 170179 belongs to a separate species.

## ANNOUNCEMENT

Staphylococcus haemolyticus is a Gram-positive coagulase-negative (CoN) staphylococcal species that is found on normal human skin. It is regarded as an important nosocomial pathogen and is associated with infections such as endocarditis and sepsis and, in particular, implant-associated infections (1, 2).

Here, we present the draft genome sequence of the CoN staphylococcal strain 170179, which was isolated from healthy human facial skin in Denmark. Primary isolation from forehead skin was achieved by a cotton swab, moistened in sampling buffer (0.1% Triton X-100 in 0.075 M phosphate buffer [pH 7.9]). The inoculated 5% blood agar plate (SSI, Denmark) was cultivated for 24 h at 37°C under aerobic conditions. Blood agar growth showed that the hemolytic property of strain 170179 was similar to that of strains of *S. haemolyticus* (1).

Genomic DNA isolation was done using the MasterPure DNA purification kit (Epicentre). The concentration and purity of the isolated DNA was checked with a NanoDrop 1000 spectrophotometer (ND-1000; Peqlab, Erlangen, Germany), and the exact concentration was determined using the Qubit double-stranded DNA (dsDNA) high-sensitivity (HS) assay kit as recommended by the manufacturer (Life Technologies, Darmstadt, Germany). Illumina shotgun libraries were prepared using the Nextera XT DNA sample preparation kit and subsequently sequenced on a MiSeq system with the reagent kit version 3 with 600 cycles (Illumina, San Diego, CA, USA) as recommended by the manufacturer. Quality filtering using Trimmomatic version 0.36 (3) resulted in 3,804,436 paired-end reads with an average read length of 232 bp. The assembly was performed with the SPAdes genome assembler software version 3.13.0 (4). The assembly resulted in 48 contigs (>500 bp) with an *N*_50_ value of 212,499 bp and an average coverage of 334-fold. The assembly was validated and the read coverage determined with QualiMap version 2.2.1 (5). Throughout, default parameters were used for all bioinformatics. The draft genome of *Staphylococcus* sp. 170179 has a size of 2.629 Mb with an overall GC content of 33.6%, thus slightly higher than that of strains of *S. haemolyticus* (on average, 32.8%).

Gene prediction and annotation were done with RAST (6); 2,533 coding sequences were predicted. Comparison of strain 170179 with closed genomes of *S. haemolyticus* strains revealed an average nucleotide identity (ANI) of only 87.5%. Based on this low ANI, we propose that this strain belongs to a separate species. A phylogenomic analysis with all strains sequenced so far that are currently classified as *S. haemolyticus* (*n* = 263) confirmed that strain 170179 belongs to a novel species; only three genomes of yet-undescribed strains were similar to the genome of strain 170179. The highest ANI (99.6%) was found with strain 51-48 (GenBank accession no. CUEE00000000), a blood culture isolate from Norway; an ANI of 97.8% was found with two strains, SNUC 119 (QYJM00000000) and SNUC 1342 (PZIO00000000), both isolated from Bos taurus in Canada (7). Genomic differences with *S. haemolyticus* exist, such as the presence of a urease gene cluster in the *Staphylococcus* sp. 170179 genome. The genome sequence of this new staphylococcal species may contribute to our understanding of the evolution of human-associated *Staphylococcus* spp.

### Data availability.

The genome sequence of *Staphylococcus* sp. 170179 has been deposited in GenBank under accession no. WOFX00000000. The raw reads have been deposited in the NCBI SRA database under accession no. SRX7263450.
